# The Crux of the Medicine Prices' Controversy in Pakistan

**DOI:** 10.3389/fphar.2017.00504

**Published:** 2017-08-02

**Authors:** Kah S. Lee, Adnan Shahidullah, Syed T. R. Zaidi, Rahul P. Patel, Long C. Ming, Muhammad H. Tariq, Obaidullah Malik, Muhammad J. Farrukh, Ahmad Khan, Siew M. Yee, Tahir M. Khan

**Affiliations:** ^1^Pharmacy, School of Medicine, University of Tasmania Hobart, TAS, Australia; ^2^Department of Pharmacy, University of Peshawar Peshawar, Pakistan; ^3^School of Pharmacy, KPJ Healthcare University College Nilai, Malaysia; ^4^School of Pharmaceutical Sciences, Universiti Sains Malaysia Gelugor, Malaysia; ^5^Drug Regulatory Authority of Pakistan Islamabad, Pakistan; ^6^Faculty of Pharmaceutical Sciences, UCSI University Kuala Lumpur, Malaysia; ^7^Department of Pharmacy, Faculty of Biological Sciences, Quaid-i-Azam University Islamabad, Pakistan; ^8^Faculty of Pharmacy, SEGi University Kota Damansara, Malaysia; ^9^School of Pharmacy, Monash University Sunway City, Malaysia

**Keywords:** consumer price index, drug costs, delivery of health care, health services accessibility, out-of-pocket expenditures

## Chronicle of medicine pricing control

Drugs are regulated in Pakistan under the Drug Act 1976 and DRAP Act, 2012, under which the sales, storage, and distribution of drugs are regulated at provincial level while the manufacturing (licensing), registration, pricing, import, export, and monitoring of controlled drugs comes under the domain of federal government. The Drug Regulatory Authority of Pakistan (DRAP) established under the DRAP Act 2012 works under the federal government to regulate the aforementioned matters including fixation of prices. Prices are fixed by the Federal government under Section 12 of Drugs Act, 1976 after a recommendation of the Drug Pricing Committee (DPC) constituted the Statutory Regulatory Orders (SRO) on 6^th^ August 2013 under the Cost and Pricing Division, DRAP. DPC is comprised of representatives from provincial health departments, Ministry of Finance and consumer bodies along with stakeholders as observers to proceedings of committee.

In retrospective, the Pakistani government, particularly Drug Regulatory Authority of Pakistan (DRAP) in collaboration with provincial health departments are responsible for regulating the medicine prices and has taken various regulatory measures to address the issue of medicines accessibility, particularly on the medicines affordability and availability. Since 2001, there is a moratorium on price increase on 821 and 108 medicines through statutory regulatory orders SRO-100, SRO-328, respectively, before the establishment of DRAP. There are media reports that between June and August 2016, DRAP has approved price increases for four times but pharmaceutical companies were reported to have increased prices for at least five times (Chaudhry, 2016) but these reports are misleading. During the price moratorium, drug prices have not been revised despite of multifactorial burdens including increase in dollar exchange rate, fuel prices, inflation, material costs. Only with the exceptional cases of failure of a pharmaceutical manufacturer to continue manufacturing at the fixed price and accessibility of that drug was not ensured for general public. Moreover, some pharmaceutical companies increased prices of their medicines and were able to get stay orders from a provincial High Court to keep their price increased until the matter was resolved.

DRAP's statutory power to regulate medicine prices were heavily opposed by the pharmaceutical industries who struggle to optimize their revenues due to limited wholesale mark-ups, ranging 2% (Cameron et al., 2009) to 10% (Mendis et al., 2007). It was getting practically non-viable for many companies including the multinationals to market their products in the same price as approved in 2001. These factors also led to stock-outs of essential medicine in healthcare institutions, especially public-funded hospitals. As a solution, to ensure the sustainability of local pharmaceutical industries and the accessibility of medicines, the first ever comprehensive Drug Pricing Policy 2015 was introduced (Drug Regulatory Authority of Pakistan, 2015). This new policy has laid down a transparent mechanism for fixation and price adjustment with an objective to help increasing availability of drugs at rational prices and discourage hoarding. Moreover, DRAP has also devised a monitoring mechanism with the coordination of the provincial health authorities working under the Provincial Quality Control Board to ensure that drugs are not sold in the market at prices higher than the approved range. According to this policy, prices of new drugs shall be fixed on the basis of average prices in India and Bangladesh and if new drug is not available in these countries price shall be fixed at the lowest level of the developing countries which regulate drugs prices or wholesale prices in UK, Australia, New Zealand. Moreover, a new concept of price reduction up to 30% on originator brands has been introduced with three staggered annual decrements.

Drug Pricing Policy 2015 links the annual increase in medicine prices with the Consumer Price Index (CPI) as announced by Pakistan Bureau of Statistics, Government of Pakistan, with a maximum cap of 4% for scheduled drugs and 6% for non-scheduled drugs. For 2016, the proposed price increase in scheduled drugs and non-scheduled drugs was merely 1.43% and 2.00% respectively, based on CPI. It must be noted that the price hike is in fact unlawful as it had not been approved by Pakistani Federal Government.

## Balance between affordability and profitability

Freezing of medicine price does not entirely address the issue of medicines accessibility. Given that 13% of the population in Pakistan lives below the international poverty line of US$ 1.0 per day, even simple analgesics such as aspirin can be unaffordable for the low-income group for long-term consumption. A study confirmed that even back in 2007, post-implementation of price freeze, medicines were generally not affordable for blue-collar workers: 1.7–7.7 days' wages (for generics medicines), and 1.9–36.4 days' wage (for brand-name medicines) were needed to purchase a 1-month supply of medication for certain chronic diseases (Mendis et al., 2007).

Some scholars and news reports have brought up the issue of high medicine price in Pakistan (Junaidi, 2014, 2016; Saleem et al., 2016). These reports claimed that the prices of medicines have been increased by more than 100%. Inadvertently, DRAP is being positioned in a tight spot due to its delicate role in balancing between ensuring medicine accessible to the public and the sustainability of pharmaceutical industries. In addressing the issue of medicine price, one should understand its multifactorial process, including the importation cost of raw and packaging materials, direct and indirect manufacturing costs, transportation and port charges, payment of taxes and duties, etc. In Pakistan, the CPI, which denotes the cost of goods, has increased more than 230% over the last 15 years. Moreover, a depreciation of over 70% in the Pakistani currency (rupee) against US dollar over the same period has hugely raised manufacturers' operational costs, having a domino incremental effect across the medicine supply chain.

As mentioned in the previous section, Pakistani Drug Pricing Policy, introduced in 2015, authorized the implementation of medicine price fixation (Drug Regulatory Authority of Pakistan, 2015). It must be noted that fixing prices for medicines by regulators is commonly practiced elsewhere. However, in any price control regulation, the most difficult step is in the determination of the fair price for each medicine. For instance, South African pharmaceutical manufacturers are required to sell their medicines at only one price, known as the maximum single exit price (SEP) which determined by the Pricing Committee. Medicine prices are revised annually based upon the CPI to better reflect the cost of manufacturing. In the initial stage of implementation in 2004, the SEP was set based on the average 2003 prices of medicine. In the case of Pakistan, the price increase moratorium from December 2001 till June 2016 without taking into consideration the annual CPI has rattled the pharmaceutical demand and supply equilibrium, creating a shortage of essential medicine in the market (Zaidi et al., 2013). Therefore, the corrective measure taken by the DRAP to link the annual increase in medicine prices with the CPI of the immediately preceding financial year is viewed as a positive move to address this conundrum.

In addition, it is vital to determine the starting point of the base cost before mark-up are added to the final retail price. Pakistan applied external reference pricing by benchmarking with countries such as India, Bangladesh, UK, Australia, and New Zealand, with a mark-up of 35 and 15% for the wholesaler and the retailer, respectively. However, several consumer advocates have claimed that Pakistan medicine price remains high and unaffordable. Therefore, it is imperative to revise the choice of countries as reference countries and the methodology used. Referencing to incorrect choice of reference countries, particularly using high-income countries and countries with different market structure may lead to high medicine prices.

Before advent of Drug Pricing Policy 2015, allegations of malpractices and mismanagement in DRAP were reported, notably after taking cognizance against Drugs Pricing Committee, DRAP by the National Accountability Bureau (NAB) along with eight accused, and directors of several pharmaceutical companies. They were charged of approving illegal and unjustified drugs price hike of these particular pharmaceutical companies (Yasin, 2016) based on controversial documents submitted by these pharmaceutical companies. Therefore it is vital for DRAP to improve the transparency in the methods and processes under which medicine prices and other functions would be regulated, in order to regain stakeholder's trust and confident. On the positive note, Drug Pricing Policy 2015 abolishes discretions in deciding medicine prices. Similarly, DRAP website (http://www.dra.gov.pk/) features a spreadsheet with a clear formula to calculate the annual medicine prices based on the CPI. The automatic price revision without the interaction with DRAP officials will reduce the likelihood of corruption and increase the accountability of DRAP.

Inevitable, DRAP and provincial health departments, without much surprise, could not monitor effectively medicine pricing in Pakistan because the ratio of population: officer is a far cry from the 2,000:1 ratio recommended by the World Health Organization (Table 1). Regulation of price control without the adequate enforcement is not feasible. Thus, in 2016 DRAP has appointed about 192 new regulators and doubled number of Pakistani federal inspector of drugs in 2017. Similarly, respective provincial governments had increased their human resources in the field of drug regulation. All these measures undertaken by DRAP and provincial health departments facilitate law enforcement and keep corruption at bay. However, further increase in human resource with proper training is needed to ensure availability of safe, effective, and quality medicines at affordable price. Basic training programs must be established to train officers extensively in law, investigation, evidence handling, and customer service.

**Table 1:** The ratio of population: drug enforcement officer in Pakistan.

**Provinces/Territories**	**Population (million)**	**Number of drug enforcement officer (full time) in 2017**	**Ratio population: officer**	**Difference in actual and WHO recommended ratio (times)**
Baluchistan	7.9	PDIs^*^ 57	136,448:1	68.2
		FIDs^**^ 01		
Punjab	101.1	PDIs 159	604,790:1	302.3
		FIDs 08		
Sindh	42.4	PDIs 27	1,211,428:1	605.7
		FIDs 08		
Khyber Pakhtunkhwa	28.2	PDIs 27	1,000,000:1	500
		FIDs 01		
Gilgit–Baltistan	1.8	PDIs 08	200,000:1	100
		FIDs 01		
FATA	3.2	PDIs (Nil)^***^	Nil	–
		FIDs (Nil)		
Azad Kashmir	4.6	PDIs 08	507,777:1	253.8
		FID 01		
Islamabad Capital Territory	1.15	PDI 01	287,500:1	143.7
		FIDs 03		

* *Provincial Drug Inspectors*.

** *Federal Inspector of Drugs*.

*** *Federally Administered Tribal Areas (FATA) Drug inspectors (Total of seven appointments under process)*.

## Medicine pricing in Pakistan: the way forward

The impact of medicine price regulation on the availability and affordability of essential medicines should be monitored regularly. Affordability study and pricing survey, to date are still lacking in Pakistan. The national affordability and pricing survey conducted in 2006 (Network for Consumer Protection, 2006), using WHO standardized methods provides comprehensive information on pricing in both the public and private sectors. Apart from this, there is little research on pricing, and no attempt at periodic updating of information.

However, to effectively contain medicine prices, a combination of different pharmaceutical pricing policies influencing the demand and supply should be adopted. Pakistani policy makers should select policies that are aligned with the objectives and suit the context of Pakistan healthcare system. As newer and more expensive medicines become available, policy makers can consider adapting other price control policies such as manage entry agreement and value based pricing. Pakistan's fixed price regulation can be relatively inflexible and may fail to take into consideration the fluctuating market dynamics. It is high time for the fine tuning of medicine price control regulation in Pakistan in light of national requirements, international best practices, and guidelines of international health partners. DRAP effectiveness in implementing these policies is crucial in ensuring the compliance of these policies, along with the presence of transparent pricing policies, process, and decisions.

In summary, it is a good sign that the health expenditure of Pakistan has increased from US$ 13.5 per capita per year in 2001 to US$ 36.2 per capita per year in 2014; yet the publicly funded healthcare facilities that supply free medication can only cater for one-fifth of Pakistan's population. To overcome this, the government of Pakistan launched National Health Insurance scheme on 31^st^ December 2015 in 23 districts for around three million lower income families (Abbasi, 2015). Through the proposed insurance scheme offered in both public and private sector hospitals, the needy would receive subsidy and compensation as per the terms and condition outlined by the insurance providers (Goverment of Pakistan, 2015). Essentially, a balance needs to be struck between affordability (for the healthcare authorities) and profitability (for the pharmaceutical industry) to ensure the growth of both pharmaceutical manufacturing and affordable medicine pricing.

## Author contributions

Conceived the conceptual framework: KL, AS, SZ, RP, LM, and TK. Wrote the paper: KL, AS, LM, MT, OM, MF, AK, and TK. Designed search strategies: KL, AS, LM, MT, AK, SY, and TK. Critically reviewed the manuscript for important intellectual content: KL, AS, SZ, RP, LM, MT, OM, MF, AK, SY, and TK. All authors read and approved the final version: KL, AS, SZ, RP, LM, MT, OM, MF, AK, SY, and TK.

### Conflict of interest statement

The authors declare that the research was conducted in the absence of any commercial or financial relationships that could be construed as a potential conflict of interest.
